# Aging Enhances Serotonergic Signaling via 5‐HT_7_ Receptors Underlying Mechanical Alloknesis

**DOI:** 10.1111/acel.70633

**Published:** 2026-07-13

**Authors:** Qiaofeng Zhao, Alberto Leguina‐Ruzzi, Mitsutoshi Tominaga, Yayoi Kamata, Atsuko Kamo, Huiying Wan, Bin Yin, Yuping Ran, Kenji Takamori

**Affiliations:** ^1^ Juntendo Itch Research Center (JIRC), Institute for Environmental and Gender‐Specific Medicine Juntendo University Chiba Japan; ^2^ Faculty of Medicine Universidad Andrés Bello Santiago Chile; ^3^ Graduate School of Health Care and Nursing Juntendo University Chiba Japan; ^4^ Department of Dermatology, Sichuan Provincial People's Hospital, School of Medicine University of Electronic Science and Technology of China Chengdu China; ^5^ West China School of Medicine Sichuan University, Sichuan University Affiliated Chengdu Second People's Hospital Chengdu China; ^6^ Department of Dermatology, West China Hospital Sichuan University Chengdu China; ^7^ Department of Dermatology Juntendo University Urayasu Hospital Chiba Japan

**Keywords:** 5‐HT_7_ receptor, aging, chronic pruritus, dorsal root ganglion, mechanical alloknesis, serotonin signaling, spinal dorsal horn

## Abstract

Chronic pruritus (or itch) is a common condition associated with aging; however, the neural mechanisms driving age‐related itch hypersensitivity remain largely unknown. Here, we investigated the role of serotonergic signaling in modulating itch sensitivity during aging. Aged mice exhibited enhanced mechanical alloknesis without changes in spontaneous scratching behavior, indicating that aging selectively affects mechanically evoked itch. Consistent with this phenotype, aged mice showed elevated urinary serotonin levels and increased expression of the serotonin receptor 5‐HT_7_ in dorsal root ganglion neurons and the spinal dorsal horn. Pharmacological blockade of 5‐HT_7_ receptors attenuated itch‐related behaviors, particularly mechanical alloknesis, indicating that serotonergic remodeling contributes to age‐related itch hypersensitivity. These findings identify spinal 5‐HT_7_ signaling as a potential therapeutic target for chronic pruritus in older individuals.

## Graphical abstract

Aging selectively enhances mechanical alloknesis. This is associated with altered systemic serotonin homeostasis and increased 5‐HT_7_ receptor expression in dorsal root ganglion neurons and spinal dorsal horn (laminae III–IV). Pharmacological inhibition of serotonergic signaling attenuates itch behavior.

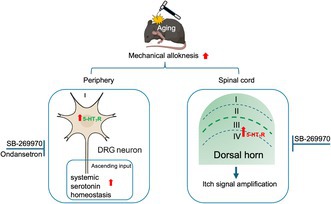

## 1 Introduction

Chronic pruritus is a common and often debilitating symptom that significantly impairs quality of life, particularly in older adults. Epidemiological studies indicate that the prevalence of chronic itch increases with age, affecting a substantial proportion of individuals over 65 years old and often persisting for years with moderate to severe intensity (Gunther et al. 2025). Clinical observations suggest that pruritus in elderly populations is frequently multifactorial and may arise from dermatologic, systemic, neurological, or idiopathic causes. Importantly, aging itself has been proposed as an independent contributor to itch susceptibility, potentially through age‐related changes in skin barrier function, immune responses, and neural signaling pathways (Chung et al. 2020). Despite the high prevalence and clinical burden of pruritus in the elderly, the underlying neurobiological mechanisms that drive age‐related itch hypersensitivity remain poorly understood.

Recent advances in neurobiology have demonstrated that itch is mediated by dedicated peripheral sensory neurons and spinal circuits that process pruritic signals independently of pain pathways (Han and Dong 2014). Primary afferent neurons located in dorsal root ganglia (DRG) detect pruritogens and relay signals to the spinal dorsal horn, where distinct neuronal populations integrate and transmit itch information to higher brain regions (Albisetti et al. 2019). Within the dorsal horn, laminar neuronal circuits coordinate the transmission and modulation of itch signals, highlighting the importance of spinal processing in itch perception. Dysregulation of these peripheral and central circuits can lead to pathological itch states, including chronic pruritus and itch sensitization (Chen 2021).

Among the neurotransmitters implicated in itch signaling, serotonin (5‐hydroxytryptamine; 5‐HT) has emerged as a key modulator of pruritic sensory transmission. Serotonin can act as a potent pruritogen and modulate the excitability of primary sensory neurons through activation of multiple receptor subtypes expressed in peripheral and central nervous system structures (Domocos et al. 2020). In addition to its well‐known role in mood regulation, serotonin is released by platelets, mast cells, and immune cells during inflammatory responses and can directly influence itch and pain signaling pathways. Experimental studies have shown that serotonergic signaling can evoke scratching behavior and modulate neuronal excitability in DRG neurons involved in sensory processing (Domocos et al. 2020).

Among serotonergic receptors, the 5‐HT_7_ receptor has recently been identified as a critical mediator of itch signaling. Genetic and pharmacological studies demonstrated that activation of the 5‐HT_7_ receptor promotes itch behavior through downstream signaling pathways that enhance sensory neuron excitability, whereas receptor blockade reduces scratching responses in experimental models (Morita et al. 2015). These findings suggest that 5‐HT_7_‐dependent serotonergic signaling represents a key pathway in both acute and chronic itch conditions. Other serotonin receptor subtypes, including 5‐HT_3_ receptors, have also been implicated in pruriceptive processing, further highlighting the complexity of serotonergic modulation of itch circuits (Miyahara et al. 2021).

Despite these advances, the contribution of serotonergic signaling to age‐related itch hypersensitivity remains largely unexplored. Aging is known to alter multiple neurotransmitter systems and neuronal circuit functions, including those involving monoaminergic signaling pathways. Changes in serotonin metabolism and receptor expression may therefore influence sensory processing and contribute to altered itch perception during aging (Lee and Kim 2022). However, the cellular and anatomical mechanisms through which aging affects serotonergic itch signaling have not been fully characterized. A growing amount of evidence indicates that aging is accompanied by neuronal plasticity and sensory circuit remodeling, resulting in altered processing of peripheral sensory inputs and increased susceptibility to maladaptive sensory phenotypes, including chronic itch (Marzola et al. 2023).

Animal models have become essential tools for investigating the neural mechanisms underlying pruritus (Han and Dong 2014). Because itch perception cannot be directly measured in animals, behavioral assays such as spontaneous scratching and mechanically evoked itch responses provide reliable proxies for pruritic sensation. Experimental models allow the integration of behavioral analyzes with molecular and neuroanatomical approaches, enabling the identification of neural circuits and signaling pathways involved in itch processing. In particular, rodent models have been instrumental in dissecting the roles of spinal circuits, neurotransmitters, and receptor systems in itch transmission and modulation (Ralvenius et al. 2018).

Aging is frequently accompanied by chronic pruritus, yet the underlying neural mechanisms remain poorly understood. Serotonergic signaling has been implicated in itch modulation; however, its role in age‐related alterations of itch circuits remains unclear. In this study, we investigated whether aging reshapes serotonergic signaling within peripheral and spinal itch pathways, using complementary behavioral, biochemical, immunofluorescence imaging, and pharmacological approaches. We hypothesized that enhanced 5‐HT_7_ receptor‐mediated signaling contributes to mechanical alloknesis during aging.

## 2 Materials and Methods

### 2.1 Animals

Male C57BL/6J mice aged 8–16 weeks (young) and 68–80 weeks (aged) were obtained from Oriental Yeast Co. Ltd. (Tokyo, Japan). Mice were housed under a 12‐h light/dark cycle at a controlled temperature of 22°C–24°C, with *ad libitum* access to food and water, in the experimental animal facility at Juntendo University Graduate School of Medicine.

All experimental procedures were approved by the Animal Care and Use Committee of Juntendo University Graduate School of Medicine and were conducted in accordance with the National Institutes of Health Guide for the Care and Use of Laboratory Animals. Every effort was made to minimize animal discomfort and distress.

### 2.2 Behavioral Assays

Scratching behavior was recorded using the SCLABA‐Next System (Noveltec Co., Kobe, Japan). Mice were acclimated to the observation chamber for at least 30 min before behavioral testing.

Mechanical alloknesis was assessed using von Frey filaments (0.07 g or 0.16 g) applied perpendicularly to the shaved dorsal skin for up to 1 s at 5‐s intervals, for a total of 30 stimulations. The alloknesis score was defined as the number of scratching responses evoked by the filament stimulation. Behavioral assays were performed as previously described (Zhao et al. 2025).

### 2.3 Quantification of Urinary Serotonin and 5‐HIAA by ELISA

Urine samples were collected at the same time of day and stored at −80°C until analysis. Urinary serotonin levels were measured using a Serotonin ELISA kit (ADI‐900‐175, Enzo Life Sciences, New York, USA), and urinary 5‐hydroxyindoleacetic acid (5‐HIAA) levels were measured using a 5‐HIAA competitive ELISA kit (EEL161, Invitrogen, CA, USA). All assays were performed according to the manufacturer's instructions.

### 2.4 Dorsal Root Ganglion (DRG) Neuron Cultures

DRG neurons were isolated from the spinal columns of C57BL/6J mice under sterile conditions and enzymatically dissociated using collagenase (LS004176, Worthington Biochemical Corporation, Columbus, OH, USA), L‐cysteine (C7352‐25, Sigma‐Aldrich, St. Louis, MO, USA), and papain (LS003126, Worthington Biochemical Corporation, Columbus, OH, USA).

The dissociated neurons were plated onto poly‐D‐lysine (PDL) (A3890401, Thermo Fisher Scientific, Waltham, MA, USA) and laminin (23017015, Thermo Fisher Scientific, Waltham, MA, USA)‐coated culture dishes or glass coverslips to promote cell adhesion. Cells were maintained in complete culture medium (CC‐4461, Lonza, Switzerland) at 37°C in a humidified incubator with 5% CO_2_ until used for experiments.

### 2.5 Immunofluorescence Staining of Cultured DRG Neurons

Cultured DRG neurons from young and aged mice were incubated for 24–36 h at 37°C in a humidified atmosphere containing 5% CO_2_. Cells were then fixed with 4% paraformaldehyde, permeabilized with 0.1% Triton X‐100, and blocked with a solution containing 2% bovine serum albumin (BSA) and 5% normal donkey serum. They were incubated with a primary antibody against 5‐HT_7_ receptor (5‐HT_7_R) (1:200; MBS9612082, MyBioSource, San Diego, CA, USA) for 2 h at room temperature or overnight at 4°C. After washing, cells were incubated with a species‐appropriate goat anti‐rabbit Alexa Fluor 488 secondary antibody (1:300; A‐11008, Invitrogen, Waltham, MA, USA) for 1 h at room temperature. Nuclei were counterstained with DAPI. Fluorescence images were captured using a fluorescence microscope (BZ‐X800, Keyence, Osaka, Japan).

5‐HT_7_R‐positive cells were quantified using Fiji software (Fiji Is Just ImageJ; based on ImageJ2, version 2.16.0/1.54p). The 5‐HT_7_R‐positive staining area relative to the nuclear area was measured. Data were obtained from 3 independent cultures, with multiple randomly selected regions (≥ 3 per dish) analyzed per culture dish.

### 2.6 Histological and Immunofluorescence Analyzes

Spinal samples were fixed in 4% paraformaldehyde, OCT‐embedded, and sectioned at 5 μm. For immunofluorescence staining, sections were blocked with 2% bovine serum albumin (BSA) and 5% normal donkey serum, followed by permeabilization with 0.1% Triton X‐100. Sections were then incubated overnight at 4°C with primary antibodies against 5‐HT_7_ receptor (1:1000; MBS9612082, MyBioSource, San Diego, CA, USA) and NeuN (clone A60, 1:1000; MAB377, Sigma‐Aldrich, St. Louis, MO, USA).

After washing, sections were incubated with species‐appropriate Alexa Fluor–conjugated secondary antibodies, including donkey anti‐rabbit Alexa Fluor 594 (1:300; A‐21207, Invitrogen, Waltham, MA, USA) and donkey anti‐mouse Alexa Fluor 488 (1:300; A‐21206, Invitrogen, Waltham, MA, USA). Fluorescence images were acquired as described above. Positive cells were manually counted. For each mouse, both sides of the spinal dorsal horn were quantified. Randomly selected fields were analyzed, and the mean value per animal was used for statistical analysis.

### 2.7 Drug Administration and Behavioral Assays

Young and aged mice were randomly assigned to treatment groups. Mice received intraperitoneal injections of one of the following compounds: SB‐269970 (5 mg/kg; S2849, Selleck Chemicals, Houston, TX, USA) or ondansetron (2 mg/kg; HY‐B0002B, MedChemExpress, NJ, USA). Immediately after injection, scratching behavior was recorded using the SCLABA‐Next System (Noveltec Co., Kobe, Japan).

Intrathecal administration was performed as previously described (Hylden and Wilcox 1980). Briefly, a 33‐gauge needle (Nipro, Osaka, Japan) was connected to a 25 μL Hamilton microsyringe (Hamilton Company, Reno, NV, USA) via approximately 30–40 cm of polyethylene tubing (PE‐10; Thermo Fisher Scientific, Pittsburgh, PA, USA) half‐filled with water. The needle tip was dipped into the drug solution, and 5 μL of solution containing either SB‐269970 (1.5 μg/5 μL) or ondansetron (1.5 μg/5 μL) was drawn into the tubing by withdrawing an equivalent volume of water using the Hamilton microsyringe. The needle was inserted into the shaved lumbar region at the L5‐L6 intervertebral space, and the drug solution was delivered intrathecally by expelling the water from the microsyringe. Successful intrathecal delivery was confirmed by characteristic tail‐flick response. Immediately after injection, scratching behavior was recorded for 1 h using the SCLABA‐Next system.

In a separate experiment, mechanical alloknesis was assessed 30 min after intrathecal administration.

## 3 Results

### 3.1 Aging Selectively Enhances Mechanical Alloknesis Without Affecting Spontaneous Scratching Behavior

**FIGURE 1:**
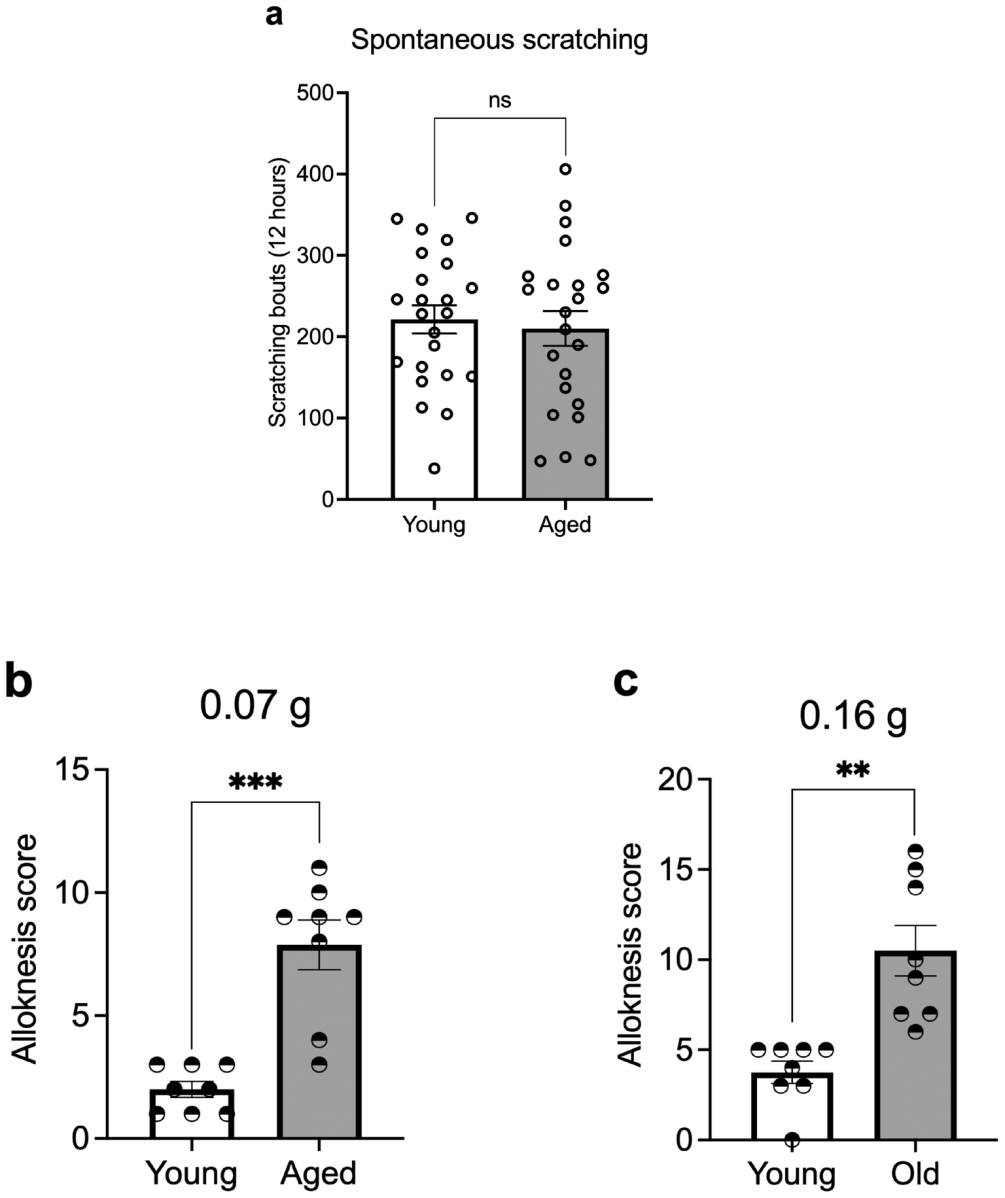
Aged mice exhibit enhanced mechanical alloknesis despite unchanged spontaneous scratching. (a) Spontaneous scratching scores were comparable between young and aged mice at 12 h (*n* = 23, ns). (b, c) In contrast, mechanical alloknesis assessed using von Frey filaments (0.07 g and 0.16 g) was significantly increased in aged mice (***p < 0.001 and **p < 0.01, respectively; *n* = 8). Data are presented as mean ± SEM. Statistical analysis was performed using Welch's *t*‐test.

To determine whether aging alters baseline itch behaviors, we first compared spontaneous scratching activity between young and aged mice. Quantification of scratching events over a 12‐h observation period revealed no significant difference between the two groups (Figure 1a), indicating that aging does not substantially alter spontaneous itch behavior under basal conditions.

We next evaluated mechanically evoked itch responses using von Frey filaments. In contrast to spontaneous scratching, aged mice displayed a marked increase in mechanical alloknesis compared with young controls. Stimulation with both low‐force (0.07 g) and moderate‐force (0.16 g) filaments produced significantly higher alloknesis scores in aged mice (Figure 1b,c).

### 3.2 Increased Urinary Serotonin Levels in Aged Mice

**FIGURE 2:**
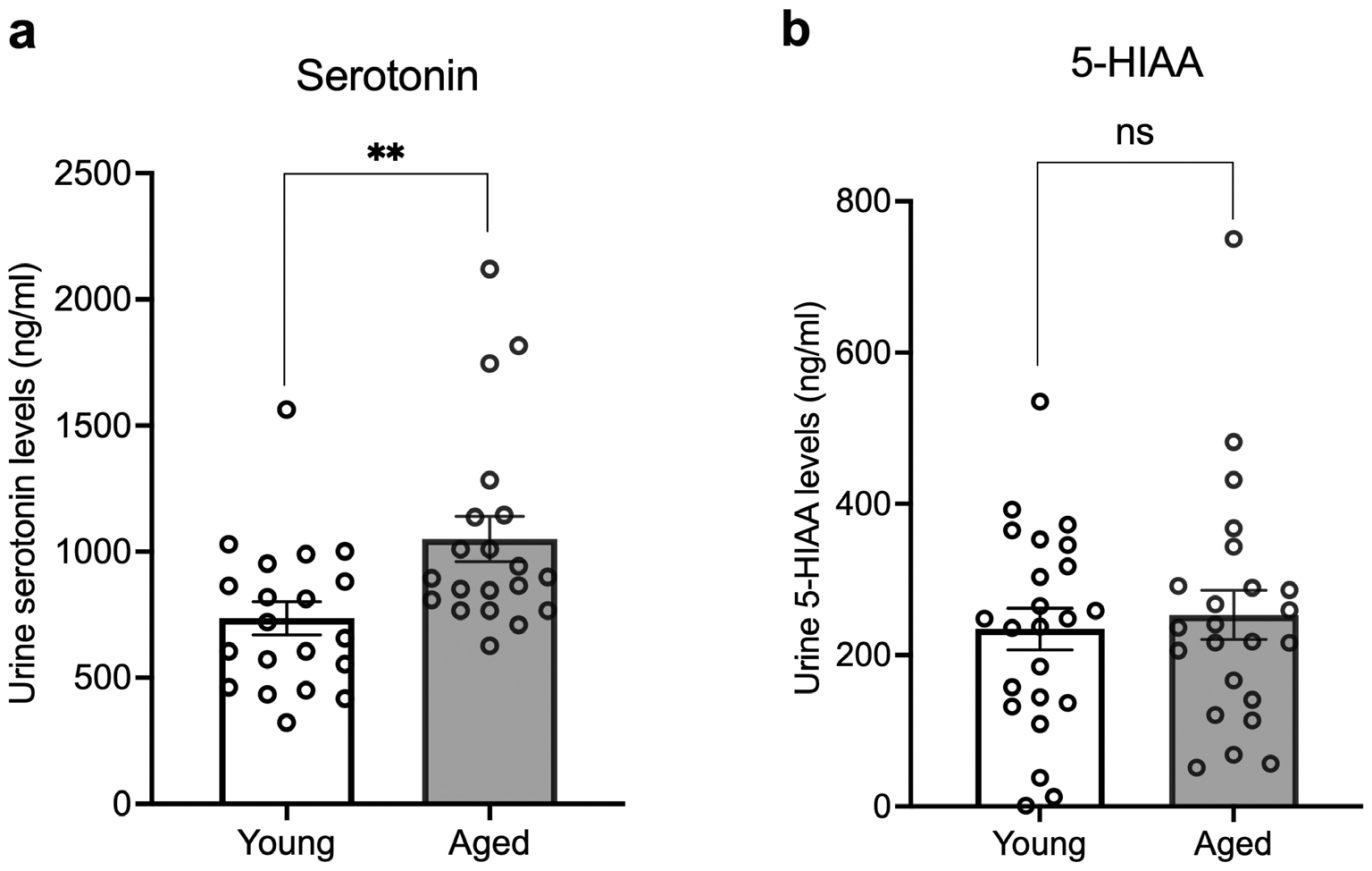
Increased urinary serotonin levels in aged mice. (a) Urinary serotonin levels were significantly higher in aged mice than in young mice (*n* = 20, **p < 0.01), whereas (b) urinary 5‐HIAA levels did not differ significantly between the two groups (*n* = 23, ns). Data are presented as mean ± SEM. Statistical analysis was performed using Welch's *t*‐test.

Given the known involvement of serotonergic signaling in itch modulation, we next examined whether systemic serotonin homeostasis is altered with aging. Measurement of urinary serotonin revealed significantly higher serotonin concentrations in aged mice compared with young controls (Figure 2a). Because urinary measurements reflect the fraction of circulating serotonin that is filtered and excreted (Berger et al. 2009; Matthes and Bader 2018), this increase likely represents elevated systemic serotonin turnover or altered peripheral serotonergic regulation.

In contrast, urinary levels of 5‐hydroxyindoleacetic acid (5‐HIAA), the principal metabolic degradation product of serotonin, were not significantly different between the two groups (Figure 2b). These findings indicate that the elevated urinary serotonin observed in aged mice was not associated with a parallel increase in its major metabolite.

### 3.3 Upregulation of 5‐HT_7_ Receptors in Dorsal Root Ganglion Neurons of Aged Mice

**FIGURE 3:**
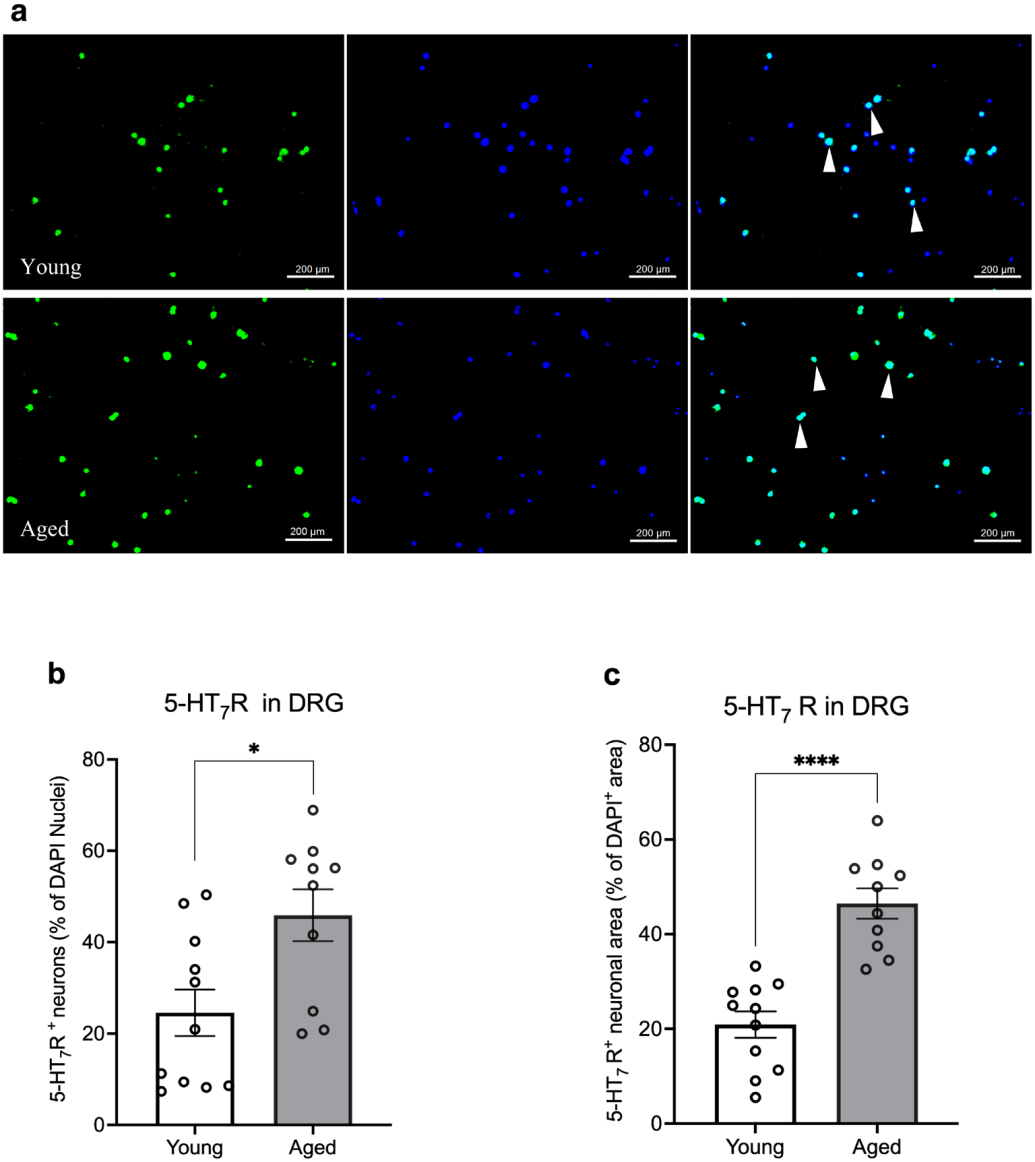
5‐HT_7_ receptor expression is increased in dorsal root ganglion neurons (DRG) of aged mice. (a) Representative immunofluorescence images of 5‐HT_7_R (green) in DRG from young and aged mice (scale bar = 200 μm). (b) Quantification of the percentage of 5‐HT_7_R‐positive neurons in the DRG. (c) Quantification of the percentage area of 5‐HT_7_R immunoreactivity in the DRG. Both were significantly increased in aged mice (*n* = 3, *p < 0.05, ****p < 0.0001). Images for quantification were captured at 4× magnification. For each mouse, at least three independent fields were randomly selected and quantified using Fiji software. Data are presented as mean ± SEM. Statistical analysis was performed using Welch's *t*‐test.

To determine whether age‐related serotonergic alterations are accompanied by changes in receptor expression within primary sensory neurons, we analyzed the distribution of serotonin receptor 5‐HT_7_ in DRG. Immunofluorescence staining revealed detectable expression of the 5‐HT_7_ receptor in DRG neurons from both young and aged mice (Figure 3a). Quantitative analysis confirmed a significant increase in the percentage of 5‐HT_7_ receptor‐positive neurons in DRG from aged mice (Figure 3b). In addition, the 5‐HT_7_ receptor‐immunoreactive area was significantly increased in aged DRG neurons (Figure 3c), indicating elevated receptor expression levels at the cellular level.

### 3.4 Age‐Dependent Increase of 5‐HT_7_ Receptor Expression in Spinal Dorsal Horn Laminae III‐IV

**FIGURE 4:**
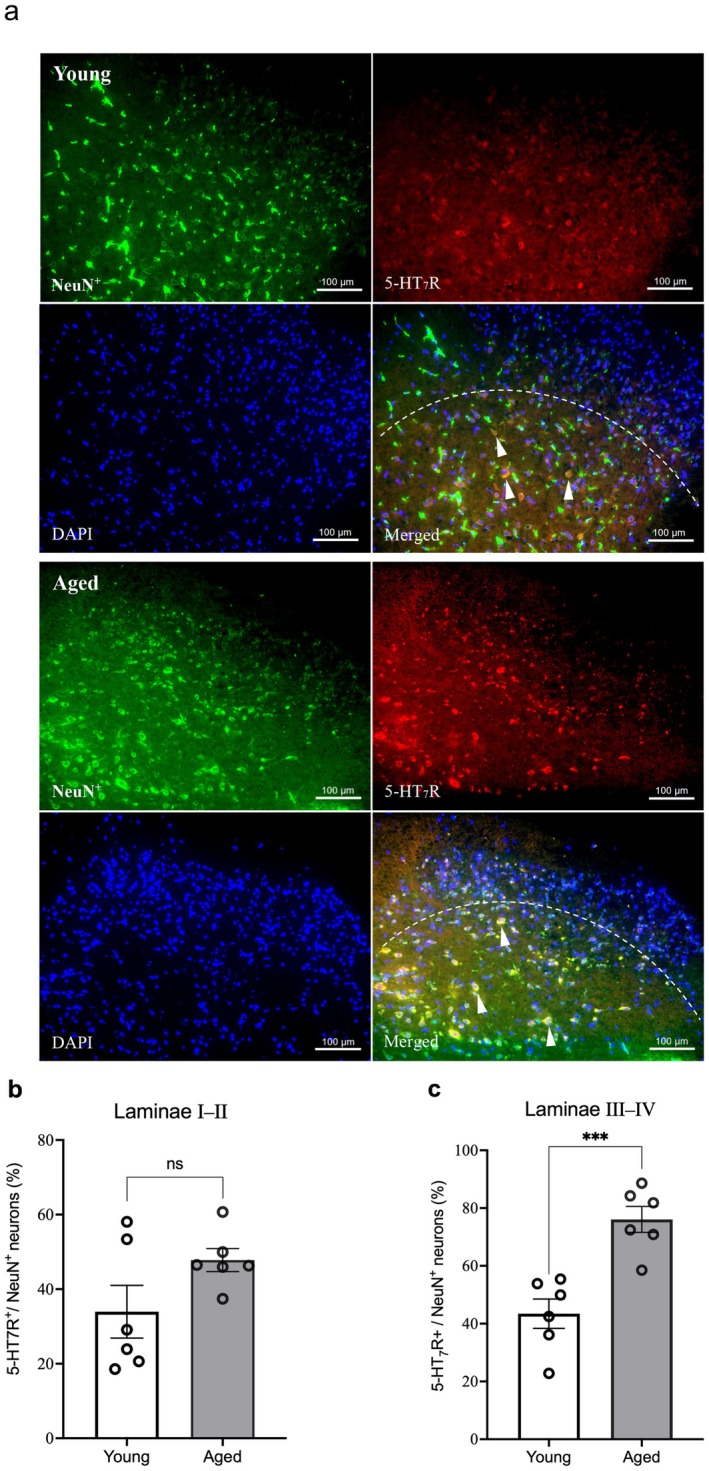
5‐HT_7_ receptor expression is increased in laminae III–IV of the spinal dorsal horn in aged mice. (a) Representative immunofluorescence images of NeuN⁺ neurons (green) and 5‐HT_7_R (red) in the spinal dorsal horn of young and aged mice (scale bar = 100 μm). (b, c) Quantification of the percentage of 5‐HT_7_R⁺ neurons (5‐HT_7_R⁺/NeuN⁺). A significant increase was observed in laminae III–IV in aged mice compared with young mice (*n* = 6, ***p < 0.001), whereas laminae I–II showed no significant difference (ns). Data are presented as mean ± SEM. Statistical analysis was performed using Welch's *t*‐test.

We investigated whether serotonergic receptor expression is also altered within spinal itch processing circuits. Immunofluorescence staining for neuronal marker NeuN and 5‐HT_7_ receptors was performed in sections of the spinal dorsal horn from young and aged mice (Figure 4a). Co‐labeling analysis allowed identification of neurons expressing the receptor within specific laminar regions.

Quantification of NeuN^+^ neurons expressing 5‐HT_7_ receptors revealed a selective increase in receptor expression within laminae III‐IV of the dorsal horn in aged mice (Figure 4b,c). In contrast, no significant differences were observed in the superficial laminae I‐II between young and aged mice.

### 3.5 Systemic Inhibition of Serotonergic Receptors Reduces Spontaneous Itch in Aged Mice

To determine whether serotonergic signaling contributes functionally to itch behavior in aged mice, we performed pharmacological inhibition of specific serotonin receptors using systemic administration. Two antagonists were used: SB‐269970, a selective 5‐HT_7_ receptor antagonist, and ondansetron, a 5‐HT_3_ receptor antagonist.

**FIGURE 5:**
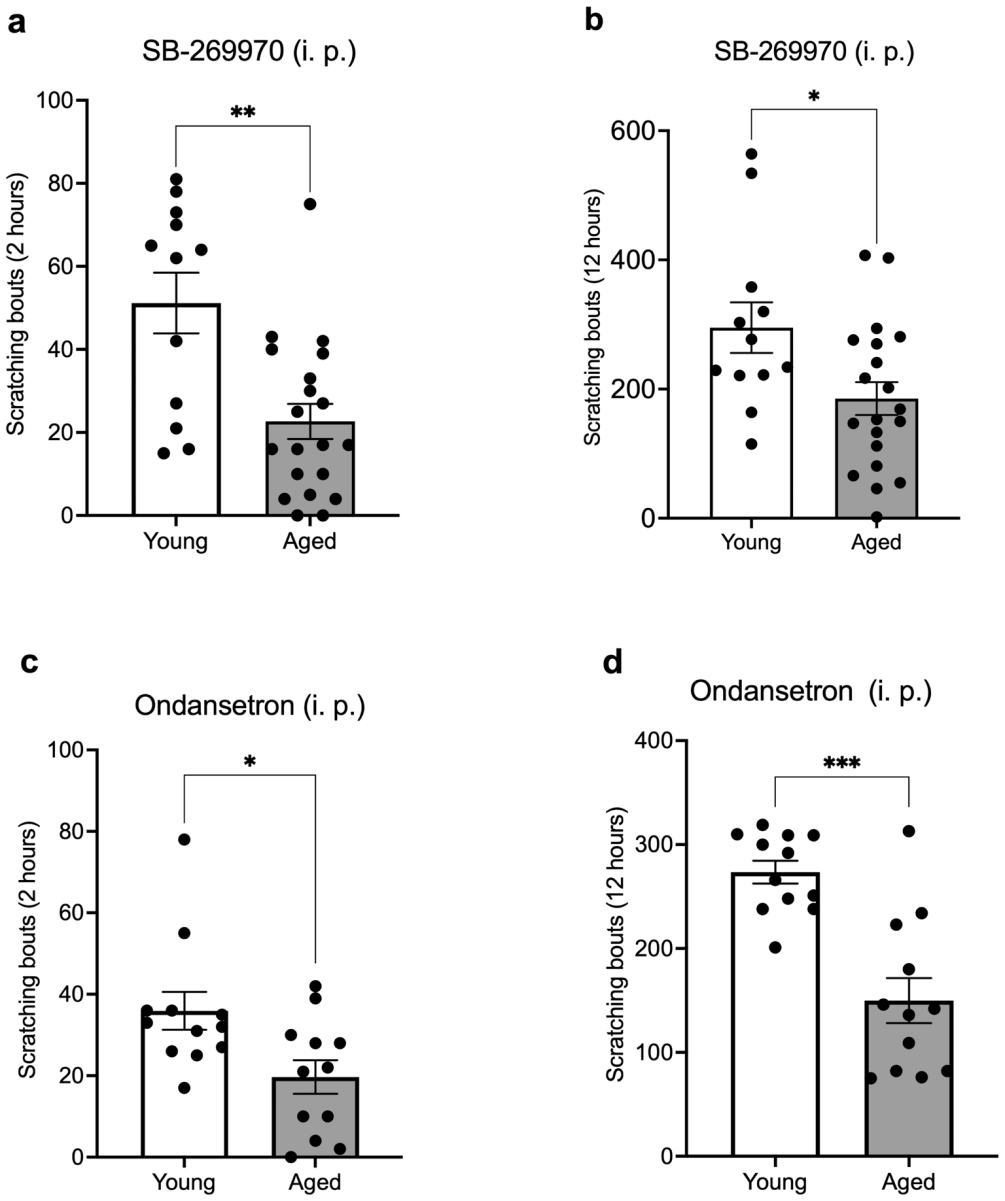
Pharmacological inhibition of serotonergic receptors reduces spontaneous itch in aged mice. Following intraperitoneal (i.p.) administration of SB‐269970 (a 5‐HT_7_R antagonist) or ondansetron (a 5‐HT_3_R antagonist), spontaneous scratching behavior was significantly reduced in aged mice. (a, b) SB‐269970 decreased scratching at 2 h (*n* = 12–20, **p < 0.01) and 12 h (*p < 0.05), whereas (c, d) ondansetron reduced scratching at 2 h (*n* = 12, *p < 0.05) and 12 h (***p < 0.001). Data are presented as mean ± SEM. Statistical analysis was performed using Welch's *t*‐test.

Intraperitoneal administration of SB‐269970 resulted in a clear reduction in spontaneous scratching behavior in aged mice at both early and later time points following treatment (Figure 5a,b). Similarly, systemic administration of ondansetron also decreased spontaneous scratching behavior in aged mice (Figure 5c,d).

### 3.6 Spinal 5‐HT_7_ Receptor Blockade Suppresses Spontaneous Itch and Mechanical Alloknesis in Aged Mice

**FIGURE 6:**
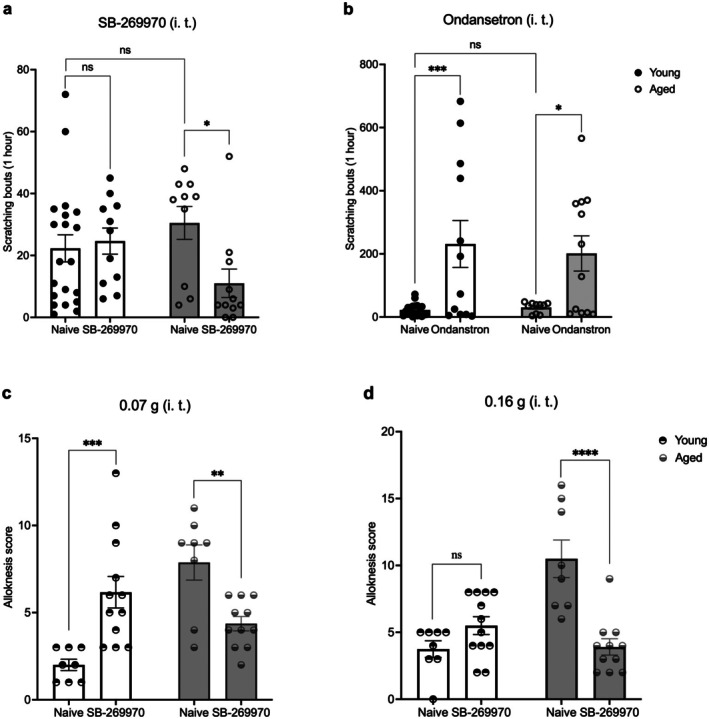
Spinal 5‐HT_7_R blockade suppresses spontaneous itch and mechanical alloknesis in aged mice. (a) Intrathecal (i.t.) administration of SB‐269970 significantly reduced spontaneous scratching in aged mice (*n* = 10–20, *p < 0.05) whereas (b) ondansetron increased scratching behavior in both young (***p < 0.001) and aged (*p < 0.05) mice during the first hour after administration. (c, d) Mechanical alloknesis was assessed using 0.07 g and 0.16 g von Frey filaments 30 min after drug administration (*n* = 8–12). SB‐269970 significantly reduced alloknesis scores in aged mice at both stimulus intensities (0.07 g, **p < 0.01; 0.16 g, ****p < 0.0001), whereas alloknesis scores were increased in young mice at 0.07 g (***p < 0.001) but showed no significant change at 0.16 g (ns). Data are presented as mean ± SEM. Statistical analysis was performed using two‐way ANOVA followed by Tukey's multiple‐comparison test.

To directly test whether spinal serotonergic signaling contributes to age‐related itch hypersensitivity, receptor antagonists were delivered intrathecally to selectively target spinal circuits. Intrathecal administration of the 5‐HT_7_ receptor antagonist SB‐269970 significantly reduced spontaneous scratching behavior in aged mice (Figure 6a). In contrast, intrathecal administration of the 5‐HT_3_ receptor antagonist ondansetron produced the opposite effect, increasing scratching behavior during the first hour following treatment (Figure 6b).

We further assessed mechanically evoked itch responses after intrathecal drug administration. Blockade of 5‐HT_7_ receptors (SB‐269970) significantly reduced mechanical alloknesis in aged mice at both (0.07 g and 0.16 g) von Frey stimulus intensities (Figure 6c,d), indicating that spinal 5‐HT_7_ signaling contributes directly to mechanical alloknesis. Notably, in young mice, SB‐269970 increased mechanical alloknesis at the lowest stimulus intensity (0.07 g), suggesting a potential age‐dependent difference in the contribution of serotonergic signaling to mechanically evoked itch processing.

## 4 Discussion

The present study found enhanced serotonergic signaling as an important mechanism contributing to age‐related mechanical alloknesis. These findings suggest that aging alters serotonergic regulation of itch pathways and promotes mechanical alloknesis through changes in both peripheral and spinal neuronal circuits (Graphical abstract).

One of the most notable findings of the present study is the increase in urinary serotonin levels in aged mice. Measurement of serotonin and its metabolites in urine is widely used as a surrogate marker of systemic serotonin turnover and peripheral serotonergic activity (Berger et al. 2009). Several analytical studies have demonstrated that urinary serotonin levels correlate with circulating serotonin and provide a reliable indicator of serotonergic metabolism in physiological and pathological conditions (Mulder et al. 2010). Quantification of urinary serotonin and its primary metabolite 5‐HIAA has long been used in clinical diagnostics, particularly for the diagnosis and monitoring of neuroendocrine tumors and other disorders associated with altered serotonergic metabolism (Kema et al. 2000). Moreover, altered urinary serotonin levels have been reported in several neurological and systemic disorders characterized by dysregulated monoaminergic signaling (Moskwa et al. 2007; Nichkova et al. 2012). In the context of aging, increased urinary serotonin may reflect alterations in systemic serotonin homeostasis, potentially due to enhanced availability or impaired clearance. Because serotonin is produced by multiple peripheral sources, including enterochromaffin cells, platelets, and immune cells, age‐related changes in these systems could contribute to altered serotonergic homeostasis (Guzel and Mirowska‐Guzel 2022). Elevated systemic serotonin may in turn increase the activation of serotonin receptors expressed on peripheral sensory neurons, thereby sensitizing pruriceptive pathways.

Serotonin has long been recognized as an important modulator of itch signaling. Experimental studies have demonstrated that intradermal administration of serotonin induces robust scratching behavior in rodents and activates pruriceptive sensory neurons (Akiyama et al. 2017; Zhao et al. 2025). Electrophysiological recordings further show that serotonergic stimulation can enhance neuronal excitability in dorsal horn circuits involved in itch processing (Zhao et al. 2014; Comitato et al. 2022). These observations are consistent with the concept that serotonin acts both as a peripheral pruritogen and as a neuromodulator of spinal itch pathways. The increased peripheral serotonin levels observed in aged mice in our study may therefore contribute to enhanced activation of serotonergic receptors within primary sensory neurons and spinal circuits. Furthermore, our results demonstrate that aging enhances 5‐HT_7_ receptor expression in primary sensory neurons. Because DRG neurons represent the first relay of peripheral sensory input, increased receptor expression may sensitize these neurons to serotonergic signaling, thereby amplifying itch‐related sensory transmission in aged mice.

Although urinary serotonin levels were increased in aged mice, urinary 5‐HIAA levels remained unchanged. Because 5‐HIAA primarily reflects serotonin degradation rather than serotonin release or receptor‐mediated signaling, unchanged 5‐HIAA levels do not necessarily exclude alterations in serotonergic signaling (Berger et al. 2009). Therefore, the elevated urinary serotonin observed in aged mice should be interpreted together with the increased 5‐HT_7_ receptor expression and the pharmacological evidence demonstrating functional involvement of 5‐HT_7_ signaling in age‐associated mechanical alloknesis.

Our immunofluorescence analyzes revealed increased expression of in spinal dorsal horn neurons located within laminae III‐IV in aged mice. This finding is particularly important because the laminar organization of the dorsal horn plays a critical role in sensory signal processing. Laminae I‐II primarily process nociceptive and pruriceptive inputs from C‐fiber afferents, whereas laminae III‐IV receive input from low‐threshold mechanoreceptors (LTMR), including Aβ fibers afferents and are involved in the processing of innocuous tactile stimuli (Comitato and Bardoni 2021). Increasing evidence suggests that mechanical alloknesis arises from abnormal interactions between low‐threshold mechanosensory pathways and spinal itch‐processing circuits within the dorsal horn, leading to the inappropriate transmission of innocuous tactile stimuli as itch signals. The selective upregulation of 5‐HT_7_ receptors observed in laminae III‐IV in aged mice therefore provides a plausible anatomical substrate for the increased mechanical alloknesis observed in our behavioral assays. Laminae III–IV receive substantial LTMR input and contain Neuropeptide Y (NPY)‐dependent inhibitory gating circuits (Follansbee and Dong 2023). These circuits normally suppress the transmission of innocuous tactile information to itch‐processing pathways. Therefore, age‐related alterations in spinal inhibitory networks may contribute to the enhanced mechanical alloknesis observed in aged mice.

Another important aspect of the present study is the functional validation of serotonergic signaling through pharmacological experiments. Systemic administration of the selective 5‐HT_7_ receptor antagonist SB‐269970 significantly reduced scratching behavior in aged mice, supporting the hypothesis that serotonergic signaling through this receptor contributes to itch hypersensitivity. SB‐269970 is widely used as a selective antagonist of the 5‐HT_7_ receptor and has been shown to modulate neuronal excitability and synaptic transmission in multiple neural circuits (Quintero‐Villegas and Valdes‐Ferrer 2022). Interestingly, intrathecal administration of SB‐269970 tended to enhance mechanical alloknesis in young mice, particularly in response to the lowest‐intensity mechanical stimulus (0.07 g), whereas spontaneous scratching was minimally affected. Because mechanical alloknesis is thought to involve abnormal processing of LTMR inputs within spinal dorsal horn circuits, these findings raise the possibility that endogenous 5‐HT_7_ receptor signaling contributes to the modulation of mechanically evoked itch under physiological conditions. In contrast, the inhibitory effect of SB‐269970 observed in aged mice suggests that aging may alter the functional role of spinal 5‐HT_7_ receptor signaling.

In contrast, inhibition of the 5‐HT_3_ receptor using ondansetron produced route‐dependent effects, reducing scratching behavior when administered systemically but increasing scratching when delivered intrathecally. These results highlight the complex and region‐specific roles of serotonergic receptors in itch modulation. Because 5‐HT_3_ receptors are ligand‐gated ion channels expressed in both peripheral sensory neurons and central interneurons, their functional effects may differ depending on the site of receptor blockade (Thompson and Lummis 2006). One possible explanation is that spinal 5‐HT_3_ receptors participate in inhibitory circuits, potentially through the activation of GABAergic and/or glycinergic interneurons (Chen and Sun 2020). Under this model, blockade of spinal 5‐HT_3_ receptors would reduce inhibitory tone and consequently facilitate itch transmission.

The potential clinical relevance of serotonergic modulation in pruritus has been explored in several studies. Ondansetron and other 5‐HT_3_ receptor antagonists have been investigated as treatments for chronic itch associated with cholestatic liver disease, uremia, and dermatologic conditions. Early clinical reports suggested that ondansetron could alleviate cholestatic pruritus, leading to rapid reductions in itch severity in some patients (Schworer et al. 1995). However, subsequent clinical trials in uremic pruritus produced inconsistent results, highlighting the heterogeneity of itch mechanisms across different disease contexts (Murphy et al. 2003). These findings suggest that targeting specific serotonergic receptor subtypes may be necessary to achieve effective therapeutic outcomes. Our results indicate that 5‐HT_7_ receptors may represent a particularly relevant target for modulating itch circuits in the context of aging.

In addition to serotonergic alterations, aging is associated with widespread changes in sensory neuron physiology and neural circuit function. Age‐related neuroinflammation, glial activation, and altered neurotransmitter signaling have been shown to influence sensory processing in both peripheral and central nervous systems. For example, aging has been linked to increased microglial activation within the spinal cord and enhanced production of pro‐inflammatory mediators that can sensitize sensory neurons (von Leden et al. 2017). Similarly, aging can alter ion channel expression and synaptic plasticity in DRG neurons, leading to changes in sensory thresholds and neuronal excitability (Jang and Garraway 2024). These neurobiological changes may interact with serotonergic signaling to promote the development of itch hypersensitivity during aging.

The interaction between serotonergic signaling and spinal itch circuits may therefore represent an important mechanism linking systemic physiological changes with altered sensory perception in elderly individuals. By enhancing receptor expression within both peripheral sensory neurons and spinal interneurons, aging may amplify serotonergic modulation of itch pathways and facilitate crosstalk between mechanosensory and pruriceptive circuits. This mechanism may be particularly relevant for explaining the high prevalence of increased mechanical alloknesis observed in elderly populations.

Several limitations should be considered. First, urinary serotonin provides only an indirect measure of systemic serotonergic activity and does not directly reflect neural serotonergic signaling. Second, although increased 5‐HT_7_ receptor expression and pharmacological blockade support a role for serotonergic signaling in age‐related mechanical alloknesis, neuronal activity was not directly assessed. Future studies using calcium imaging or electrophysiological approaches may help clarify how aging alters serotonergic modulation of itch circuits. Third, the specific spinal circuits involved remain unclear. In particular, the potential contribution of inhibitory pathways, including NPY‐dependent gating mechanisms, warrants further investigation.

In conclusion, the present study demonstrates that aging is accompanied by alterations in serotonergic signaling that contribute to enhanced mechanical alloknesis. Increased 5‐HT_7_ receptor expression and the effects of receptor blockade support a role for 5‐HT_7_ signaling in age‐related itch hypersensitivity. These findings provide new insight into the neurobiological mechanisms of age‐related pruritus and suggest that targeting spinal 5‐HT_7_ receptors may represent a novel therapeutic strategy for chronic itch in older individuals.

## Author Contributions

**Qiaofeng Zhao, Mitsutoshi Tominaga** and **Kenji Takamori:** conceptualization. **Qiaofeng Zhao, Mitsutoshi Tominaga, Yayoi Kamata** and **Atsuko Kamo:** methodology. **Qiaofeng Zhao:** data creation. **Qiaofeng Zhao, Alberto Leguina‐Ruzzi** and **Mitsutoshi Tominaga:** formal analysis. **Qiaofeng Zhao:** investigation. **Qiaofeng Zhao** and **Alberto Leguina‐Ruzzi:** writing‐original draft preparation. **Qiaofeng Zhao, Alberto Leguina‐Ruzzi, Mitsutoshi Tominaga, Yayoi Kamata, Atsuko Kamo, Huiying Wan, Bin Yin, Yuping Ran** and **Kenji Takamori:** writing‐review and editing. **Mitsutoshi Tominaga** and **Kenji Takamori:** supervision. **Qiaofeng Zhao, Mitsutoshi Tominaga** and **Kenji Takamori:** funding acquisition. All authors have read and agreed to the published version of the manuscript.

## Funding

This work was supported by Grant‐in‐Aid for Early Career Scientists (Grant 23K15292), Grant‐in‐Aid for Scientific Research (B) (Grants 23K24217 and 23K20320) and Grant‐in‐Aid for Challenging Research (Exploratory) (Grants 24k22237 and 25K22714).

## Ethics Statement

All animal experiments adhered to National Institutes of Health Guidelines for the Care and Use of Laboratory Animals. Experimental protocols were approved by the Ethical Committee of Research Animal Use of Juntendo University (Approval No. 2024104, No. 2025064).

## Conflicts of Interest

The authors declare no conflicts of interest.

## Data Availability

Data are available from the corresponding author upon reasonable request. No code was used in this study. Materials Availability: This study did not generate new unique reagents.
